# Parameters, practices, and preferences for regulatory review of emerging biotechnology products in food and agriculture

**DOI:** 10.3389/fbioe.2023.1256388

**Published:** 2023-09-28

**Authors:** Jennifer Kuzma, Khara Grieger, Ilaria Cimadori, Christopher L. Cummings, Nick Loschin, Wei Wei

**Affiliations:** ^1^ Genetic Engineering and Society Center, North Carolina State University, Raleigh, NC, United States; ^2^ School of Public and International Affairs, North Carolina State University, Raleigh, NC, United States; ^3^ Department of Applied Ecology, North Carolina State University, Raleigh, NC, United States; ^4^ North Carolina Plant Science Initiative, North Carolina State University, Raleigh, NC, United States; ^5^ Yale School of the Environment, Yale University, New Haven, CT, United States; ^6^ Engineering Research and Development Center, United States Army Corps of Engineers, Vicksburg, MS, United States; ^7^ Gene Edited Foods Project, Iowa State University, Ames, IA, United States

**Keywords:** regulation, risk assessment, governance, biotechnology, gene editing

## Abstract

This paper evaluates the U.S. regulatory review of three emerging biotechnology products according to parameters, practices, and endpoints of assessments that are important to stakeholders and publics. First, we present a summary of the literature on variables that are important to non-expert publics in governing biotech products, including ethical, social, policy process, and risk and benefit parameters. Second, we draw from our USDA-funded project results that surveyed stakeholders with subject matter expertise about their attitudes towards important risk, benefit, sustainability, and societal impact parameters for assessing novel agrifood technologies, including biotech. Third, we evaluate the regulatory assessments of three food and agricultural biotechnology case studies that have been reviewed under U.S. regulatory agencies and laws of the Coordinated Framework for the Regulation of Biotechnology, including gene-edited soybeans, beef cattle, and mustard greens. Evaluation of the regulatory review process was based on parameters identified in steps 1 and 2 which were deemed important to both publics and stakeholders. Based on this review, we then propose several policy options for U.S. federal agencies to strengthen their oversight processes to better align with a broader range of parameters to support sustainable agrifood products that rely on novel technologies. These policy options include 1) those that would not require new institutions or legal foundations (such as conducting Environmental Impact Statements and/or requiring a minimal level of safety data), 2) those that would require a novel institutional or cross-institutional framework (such as developing a publicly-available website and/or performing holistic sustainability assessments), and 3) those that would require the agencies to have additional legal authorities (such as requiring agencies to review biotech products according to a minimal set of health, environmental, and socio-economic parameters). Overall, the results of this analysis will be important for guiding policy practice and formulation in the regulatory assessment of emerging biotechnology products that challenge existing legal and institutional frameworks.

## 1 Introduction

Due to recent advancements in biotechnologies, new gene-edited food and agricultural products are now reaching the market. For example, oil from gene-edited soybeans, meat from heat-tolerant gene-edited cattle, and gene-edited mustard greens with lowered pungency have been cleared by regulatory agencies for market release, and many more gene-edited products are in late R&D stages (FDA, 2019a; Splitter, 2019; USDA, 2020b; USDA, 2020c; Erickson, 2022; FDA, 2022; Pixley et al., 2022; Mullins, 2023; USDA, 2023).

Coupled with this growth and innovation, is the evolution of the regulatory landscape of gene-edited products. Among one of the most recent changes has been the implementation of new regulations of genetically engineered organisms in the United State Department of Agriculture’s (USDA) SECURE rule (USDA, 2020a). The SECURE rule represents the most comprehensive and substantial set of changes to the oversight of genetically engineered and modified crops in the U.S. in decades. If applied as intended, the vast majority of genetically engineered crops would be exempt from premarket field testing and risk assessment requirements (Kuzma and Grieger, 2020). The SECURE rule and other regulatory oversight mechanisms for biotechnology products often involve assessments that predominantly focus on potential impacts to agriculture (USDA, “plant pest risk” under the Plant Protection Act--PPA), human health (Food and Drug Administration’s—FDA’s voluntary consultation for food under the Federal Food Drug and Cosmetic Act--FDCA), and non-target species and human health (EPA under the Federal Insecticide, Fungicide, and Rodenticide Act--FIFRA) (NASEM, 2017; OSTP, 2017; Hoffman, 2021).

While this focus on health and environmental assessments is understandable given the limited legal basis of the regulatory system and traditions of risk assessment, a broader focus of oversight may be better suited for the next-generation of agricultural biotechnologies, given the importance of wider ecosystem impacts, sustainability aspects, and associated ethical and societal implications (e.g., Kuzma et al., 2008; Kuzma, 2018; Kuzma, 2021a; Kuzma, 2021b; Kuiken et al., 2021; Rohr et al., 2021; Florin, 2022; Gould et al., 2022; Kjeldaas et al., 2022; Lindberg et al., 2023). Consumers also consider parameters of transparency, trust, choice, equitable distribution of risks and benefits, animal welfare, and longer-term ecosystem consequences to be important for their acceptance of emerging technologies and their products (Kuzma et al., 2009; Frewer et al., 2013; NASEM, 2016; PEW, 2016; Kuzma, 2021a; Cummings and Peters, 2022a; 2022b). Oversight processes and assessments that pay attention to these broader dimensions are likely needed to ensure public confidence and trust in, as well as more robust and holistic analysis of consequences of, emerging biotechnologies in food and agriculture (e.g., Kearnes et al., 2006; Kuzma et al., 2008; Kuzma et al., 2009; Hartley et al., 2016; Kuzma, 2018; Macnaghten and Habets, 2020; Kuzma, 2021b; Kershaw et al., 2021; Kjeldaas et al., 2021; Kokotovich et al., 2022).

Building off this background, this article briefly reviews the regulatory process and assessments for some of the first gene-edited agrifood products cleared for market release in the U.S. and reflects upon how these regulatory processes match up (or not) to the parameters stakeholders and publics indicate that they care about when evaluating novel biotechnologies. In particular, we evaluate the regulatory decision-making processes and assessments for three case studies involving gene-editing (oil-altered soybean, heat tolerant cattle, and less pungent mustard greens), and compare them to the parameters and practices deemed important by a range of stakeholders and consumers when evaluating novel agrifood technologies more broadly. After this review, we provide suggestions for improving the regulatory review under three categories: 1) those that would not require new institutions or legal foundations, 2) those that would require a novel institutional or cross-institutional framework, and 3) those that would require the agencies to have additional legal authorities. Overall, the results of this analysis will be important for guiding policy practice and formulation in the oversight of novel agrifood products that rely on gene-editing in order to ensure safety, consumer confidence, and positive societal impacts.

## 2 Parameters for governance important to stakeholders and consumers

In this section, we first present a summary of the literature on variables that are important to non-expert publics in governing biotech products, including ethical, cultural, social, policy process, and risk and benefit parameters. Second, we draw from our USDA-funded project results that surveyed U.S. stakeholders with subject matter expertise about their attitudes towards important risk, benefit, sustainability and societal impact parameters for assessing novel agrifood technologies, including biotech.

### 2.1 Factors important to consumers

Several studies have identified a variety of factors important to consumers regarding gene-edited foods (GEFs) and genetically modified (GM) foods that are crucial in shaping their acceptance and decision-making processes. While it is sensible to believe that people primarily make decisions about food based on cost, appearance, taste, and nutritional content, recent studies by Cummings and Peters (2022a, 2022b) show that other factors influence perceptions and levels of acceptance, including social and ethical values, trust in agricultural biotechnology companies and government, and science and technology beliefs. These factors were found to greatly influence both consumers’ willingness to eat GEFs as well as purposeful avoidance of GEF products. In addition, in these studies, individuals who are more willing to eat GEFs generally view science and technology as a primary means to solve society’s problems, they place high levels of trust in government food regulators and the agriculture biotechnology industry, and generally do not have strong beliefs about food production. These views were also associated with younger (<30) individuals with higher-than-average education and household incomes. Conversely, individuals who reported they would prefer to purposefully avoid eating GEFs are more skeptical of the value of science and technology, they place greater value on the way their food is produced, and they more readily trust environmental groups rather than government and industry. This group tends to have lower incomes, are more religious, older and female, with approximately 60% of the women surveyed reporting that they would purposefully avoid eating GEFs. Both groups agree that they would prefer that GEFs be mandated by the federal government to be labelled, with approximately 75% of the entire sampled population desiring labeling regardless of whether they would consume the products. Although the transparency of labeling is preferred by consumers, the effect of providing additional scientific information on consumer acceptance of GM foods in surveys demonstrates mixed findings. In addition, some studies report that information provision increases acceptance (Dolgopolova et al., 2017a; Dolgopolova et al., 2017b; Carrasson, et al., 2021) while others demonstrate that providing new information about GM foods does not improve consumer acceptance (Mcfadden and Wilson, 2018; Scott et al., 2018).

GEFs is also intertwined with the history of genetically modified organisms (GMOs) (Cummings, 2017; Friedrichs et al., 2019; Kuzma, 2022; Cummings et al., 2023; Lindberg et al., 2023). Public trust in GMOs has shown a significant discrepancy between scientific experts and the public. For instance, in 2015, 88% of scientists believed GMO foods were safe for human consumption compared to only 37% of the public (Pew Research Center, 2015). Recent stakeholder studies show that proponents of GEFs are seeking to cultivate public acceptance by focusing on shared values and transparency in their communication while also seeking to define GEFs as heterogeneous to GMOs (Cummings et al., 2023). Critics, on the other hand, view many of the concerns of GEFs as similar to GMOs and often seek to define GEFs as analogous to GMOs so that regulatory oversight and labeling mandates for GEFs are the same as GMOs (Cummings et al., 2023).

In a study comparing GM foods to GEFs, consumers viewed CRISPR and GM food similarly and substantially less positively than conventional food (Shew et al., 2018). Other studies show that cisgenic crops (genetic changes introduced from the same species, such as those produced by some gene-editing technologies) may be more acceptable to consumers than transgenic crops (genetic changes introduced from a different species), but that consumers may be less willing to accept cisgenic crops in comparison with conventionally bred crops (Edenbrandt et al., 2018; De Marchi et al., 2019). In Denmark, Edenbrandt et al. (2018) found a preference for cisgenic over transgenic rye bread production methods, while Marette et al. (2021) observed that French consumers would avoid gene-edited apples if given the choice. However, certain benefits associated with GEFs and GM foods can also outweigh negative perceptions among consumers, such as improved nutrition or safety (Yue et al., 2015a; Yue et al., 2015b). Furthermore, Kato-Nitta et al. (2019) found that Japanese consumers were more concerned about gene-edited livestock (pigs) than they were gene-edited vegetables (tomatoes). This study also found that the public was more willing to accept gene-edited products that provided direct-to-consumer benefits (increased nutritional value in the tomato) than products that benefited farmers (size enlargement of livestock). Only a subset of consumers reject cisgenic and transgenic crops under any circumstance (typically less than 20 percent), and other groups chose them based on health, safety and nutritional benefits, irrespective of whether they were cisgenic or transgenic (Yue et al., 2015b; Siegrist, 2008; Edenbrandt et al., 2018; De Marchi et al., 2019; Busch et al., 2022). For GM foods, benefits of increased health, safety and nutrition, particularly for those with food security needs, tend to be favored by consumers over improved taste and environmental benefits (Yue et al., 2015a). Animal welfare is another benefit from gene-edited agricultural products that can trump negative consumer perceptions. McConnachie et al. (2019) found positive consumer attitudes towards hornless gene-edited cattle, and Kilders and Caputo, (2021) found that animal welfare had the strongest positive impact on consumer willingness to purchase GM or GEF milk. In general however, other surveys show more negative attitudes towards animal gene-editing and genetic engineering than plant-based biotechnology (Frewer et al., 2014).

While ongoing studies investigate potential risks associated with GEFs, including off-target effects, unintended on-target effects, and unintended consequences (Kawall et al., 2020), scholars suggest that trust in emerging technologies for food is influenced by factors beyond technical risks and benefits, including past experiences with technology controversies, transparency and openness on the part of those who manage the technology, and provision of consumer control and choice (Slovic, 1987; Yawson and Kuzma, 2010; Kuzma and Kokotovich, 2011; Brown and Kuzma, 2013; Dietz, 2013; Yue et al., 2015a; Brown et al., 2015; Yue et al., 2015b; Cummings et al., 2023). For example, institutional trust plays a pivotal role in public perceptions and acceptance of both GEFs and GM foods (Frewer et al., 2014; Yue et al., 2015a; Cummings and Peters, 2022a; Cummings and Peters, 2022b). In summary, trust in those who manage the technology which is fostered by openness, transparency, assurance of safety, as well as consumer choice are important to consumers as well as tangible benefits that improve safety, transparency, and animal welfare when it comes to attitudes and acceptance of GEFs and GM foods by consumers.

### 2.2 Factors important to stakeholders

As a part of a USDA/NIFA-funded research project (Grant number 2022-67023-36730, PI/CoPI = Grieger/Kuzma), our research team conducted an online survey to investigate stakeholder views of parameters that would be important when evaluating novel technologies in food and agriculture, including gene editing. The approach and overview of results are provided below.

#### 2.2.1 Methods

The survey was developed using an online survey platform (Qualtrics) and was conducted anonymously with no identifying information collected. The survey consisted of 8 multiple-choice and open-ended questions to gauge respondents’ views of parameters that would be important when evaluating potential benefits and risks of novel technologies in food and agriculture (Table 1). In the multiple choice questions, participants were asked to rate the level of importance of each parameter for inclusion in benefit and risk evaluations of novel agrifoods using a 7-point semantic differential scale (1 = Not important at all, 7 = Extremely important). Participants were asked to rate the level of importance of each parameter as they were relevant for i) human health, ii) the environment, iii) animal health, and iv) ethical, legal, and societal implications (ELSI). Participants were also able to report additional parameters that they considered to be highly important to benefit and risk evaluations of novel agrifoods. The parameters included in the survey were parameters or factors included in peer-reviewed publications and based on expert knowledge of benefits and risk of novel food and agricultural technologies. These parameters were included in the survey randomly, and categories of parameters were also shown randomly; meaning the order in which the parameters were included in the survey changed between participants to avoid additional biases based on order rated by participants. The survey also asked respondents about the sector(s) in which they work and area(s) of expertise.

**TABLE 1:** List of parameters included in the stakeholder survey. Participants were asked to rate the level of importance of each parameter as they were relevant for four different categories.

Human health	Environment	Animal health	Ethical, legal, and societal implications
Food quality (e.g., taste, smell, appearance, shelf-life)	Use of environmental resources (e.g., use of environmental resources, such as water, energy, land, fisheries and wildlife resources, natural habitats)	Animal welfare and wellbeing (i.e., an animal’s condition or treatment, including physical and emotional wellbeing experienced from living conditions, disease prevalence, and/or management practices)	Food access and/or security (e.g., access to sufficient, affordable, and nutritious foods; Resiliency of food supply)
Food nutrition (e.g., nutritional value, vitamin content)	Use of agrochemicals (e.g., pesticides, herbicides, fertilizers)	Animal growth and production (i.e., an animal’s growth, development, and production, including changes in an animal’s size or weight over its lifetime)	Social justice and equity (e.g., adequate and equitable access to foods; Equitable distribution of benefits and risks of food supply; Implications for vulnerable individuals and/or communities)
Food safety (e.g., presence of pathogens, contaminants, allergens)	Impacts on climate change (e.g., emissions of greenhouse gasses, ability to sequester carbon)	Animal reproduction (i.e., an animal’s ability to reproduce and produce progeny or offspring)	Transparency (e.g., transparency in food supply, including transparency of ingredients in food and use of food labels)
Consumption patterns of nutritious foods (e.g., increased or decreased consumption rates of foods that contain essential nutrients)	Ecotoxicity (i.e., degree to which substance(s) cause harm to the environment, including impacts to living organisms, includes acute and/or chronic ecotoxicity, bioaccumulation persistence, gene transfer, and replicability)	Toxicity to animal health (i.e., degree to which substance(s) cause harm to animal health, including acute and/or chronic toxicity, allergenicity, and other adverse impacts on animal health)	Stakeholder inclusion and engagement (e.g., stakeholder participation and inclusion in development and oversight processes)
Occupational health and safety (e.g., health and safety considerations in production, use, transportation, disposal, and handling of materials and products)	Impacts on ecosystem services (e.g., nutrient cycling, pollination)		Regulations and government oversight (e.g., approval by regulatory agencies, Considered to be Generally Recognized as Safe (GRAS))
Toxicity to human health (i.e., degree to which substance(s) cause harm to human health, including acute and/or chronic toxicity, allergenicity, and other adverse impacts on health)			
Impacts on vulnerable populations (e.g., children, pregnant women, elderly)			

Study participants were identified through reviewing peer-reviewed literature, conferences, and workshops related to novel agrifood technologies. In total, we identified an initial list of 402 potential stakeholder participants from the U.S. across sectors and invited them to participate in the online survey via email. The outreach email included an overview of the survey, approximate time it would take to complete, and how information and results were handled. Before reaching out to participants, the research team submitted the survey protocol to the PI’s research institution (NC State, IRB protocol 25434), which was deemed to be IRB exempt. All study participants were able to directly access the survey using a link included in the outreach email. After the study period ended (3 weeks in the fall of 2022), the survey was closed and participants were no longer able to access the survey. Study participants were required to provide consent before responding to survey questions.

A total of 114 participants agreed to participate in the study and completed part of the survey. Out of the 114 initial study participants, only 79 participants completed the entire survey. Using the responses from the 79 participants that completed the survey, we then reviewed and cleaned the data to remove incomplete or invalid responses. This resulted in a dataset consisting of valid and completed responses from 77 participants; therefore 77 participants is considered to be the final sample size for this study. We note here that the 77 participants who completed all survey questions may not be fully representative of all 402 participants that we targeted in the original outreach and recruitment effort. Nonetheless, a final sample size of 77 is a robust sample size for social science research. Out of the 77 participants who completed the survey, more than a third of participants reported to be affiliated with academia (36.9%), followed by industry/private sector (22.62%), non-governmental organization/advocacy group (20.24%), government/public sector (11.9%), and other (8.33%). The participants also reported their areas of expertise within agriculture (23.11%), biotechnology (14.62%), nanotechnology (8.96%), ecology and/or environmental sciences (7.55%), legal or regulatory issues (7.08%), food production or processing (6.60%), life sciences (6.13%), water quality (6.13%), societal issues (5.19%), among other areas.

After the study was completed, responses were exported from the Qualtrics platform for analysis in SPSS version 28.0.0.0. For the multiple-choice questions, frequency and percentage of participant responses were calculated from the 77 participants who completed the survey. Cronbach’s Alpha reliability testing was conducted to evaluate *a priori* categorization of health and benefit parameters (e.g., human health, environment, ELSI), all categories demonstrated high reliability (alpha >.7). Further exploratory factor analysis was conducted to note possible item dimension reduction using Promax rotation and isolating factors within eigenvalues greater than one–however, these tests demonstrated similar findings to the *a priori* categories which were therefore maintained for subsequent analysis. Tests of difference were conducted using ANOVA to evaluate if there were significant differences between respondent self-reported affiliation groups (e.g., academics, industry, government, etc.). For the open-ended questions, participant responses were coded using descriptive coding processes. In this step, we reviewed participant responses, identified key themes that emerged, and assigned codes and subcodes.

#### 2.2.2 Results

**FIGURE 1:**
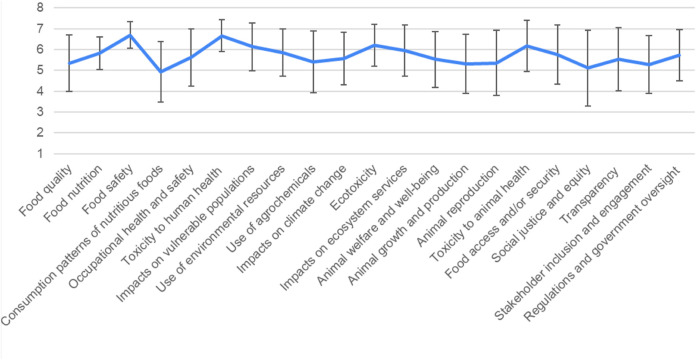
Results of stakeholder survey across all parameters, shown in mean scores and standard deviation.

First, survey results show that nearly all the investigated parameters were considered to be important by study participants, as 20 out of 21 were rated above a 5 (with ‘impacts on consumption behavior’ rated just below 5) (Figure 1). This means that stakeholders thought they were essentially all important when evaluating potential benefits and risks of novel agrifoods products. Study participants also indicated that human health and the environment were more important than animal health and ELSI-based parameters, supported by statistical tests in SPSS.

Moving from most important to least important, the most important parameters indicated by stakeholders were food safety, toxicity to human health, ecotoxicity, toxicity to animal health, and impacts on vulnerable populations, which all had mean scores above 6. The next most important parameters were impacts on ecosystem services, use of environmental resources, food nutrition, food access and/or security, regulations and government oversight, occupational health and safety, impacts on climate change, transparency, and animal welfare and wellbeing, which all had mean scores above 5.5. Following these parameters, use of agrochemicals, animal reproduction, food quality, animal growth and production, stakeholder inclusion, and social justice and equity were important, with mean scores between 5 and 5.5. Consumption patterns was the only parameter that had a mean score less than 5.

Overall, these results indicate that stakeholders consider a wide range of parameters to be important when evaluating novel food and agriculture technologies. These parameters span categories of human health, environment, animal health, as well as ELSI, and go beyond traditional parameters of human health and environmental risk and safety.

### 2.3 Parameters for evaluating case studies

The parameters in Table 1 are classified into four categories, i.e., environmental, human health, animal health, and ethical, legal and social-economic implications (ELSI). These categories also reflect the pillars of sustainability, which was popularized by the United Nations (2015) through mainstreaming sustainable development goals on a global scale (environment, health, social-economic). Agriculture and food production is one of the most challenging issues for human society regarding sustainability, given the limited natural resource capacities of the planet. Thus, in order to achieve sustainable agriculture through biotechnology, we argue that a more holistic assessment based on these parameters of sustainability should be employed before commercializing gene-edited crops and foods on a large scale in order to ensure the biotechnology products’ contribution to sustainability (see also Wei et al., 2023).

We note here that many of the parameters and their categories may likely overlap with one another and may be difficult to measure (e.g., impacts on climate change). For example, social-economic considerations address the overlapping intersections of ethical, legal, as well as economic issues that may have multiple impacts on society. Similarly, impacts on human health are also known to influence socio-economic issues, etc. In addition to intersections, the perceptions of these key parameters may also interact with one another. For example, consumer views towards human health may directly influence perceptions of food quality as well as ELSI considerations (e.g., transparency).

**FIGURE 2:**
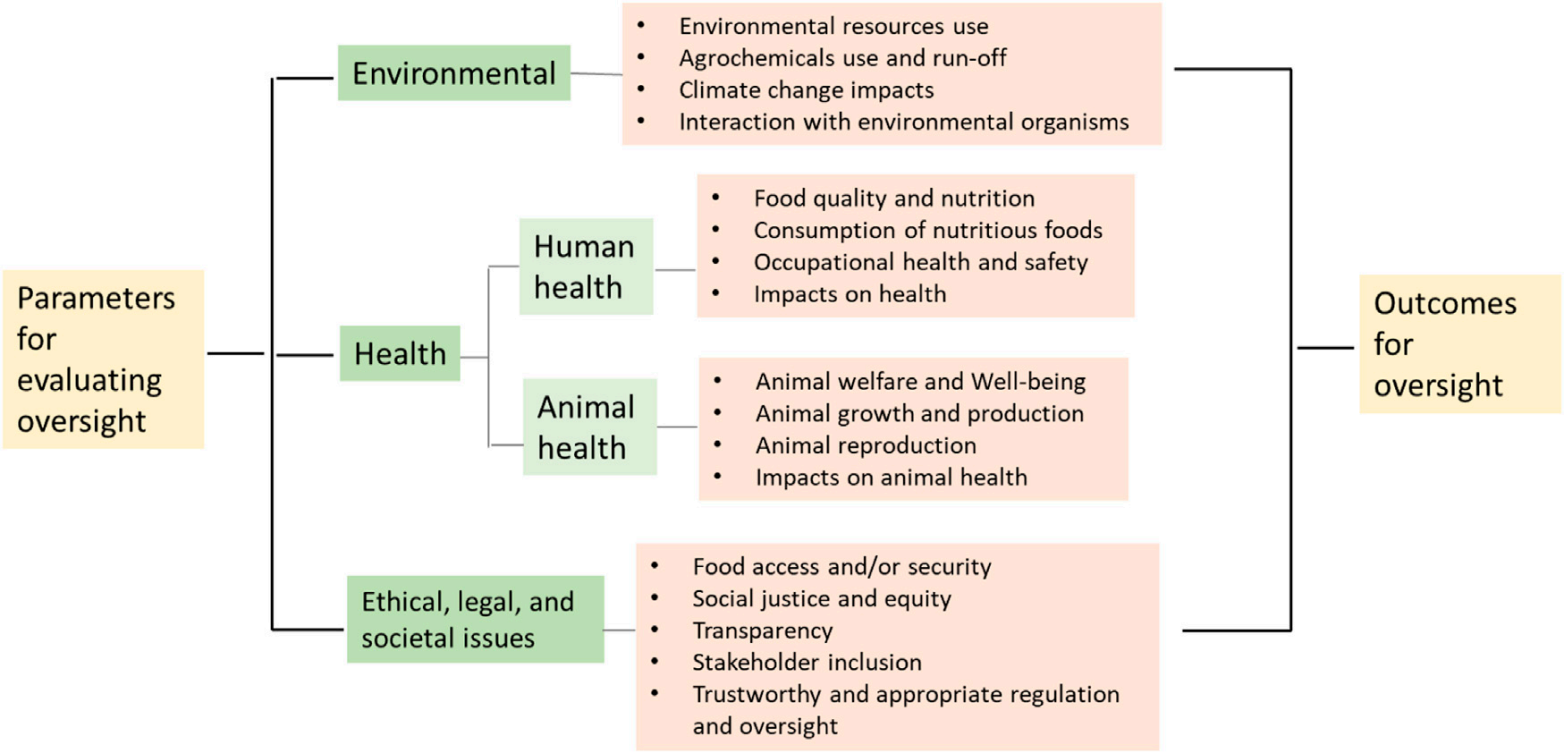
Parameters for evaluating oversight.

The parameters in Table 1 also encompass the dimensions that consumers value when it comes to acceptance of gene-edited foods (Section 2.1), including benefits such as improvements in safety and nutrition and process criteria such as transparency and openness for decision-making that creates choice for them. Thus, these parameters may serve as a set of criteria for evaluating the recent oversight of three gene-edited products in the U.S. (Figure 2).

## 3 Case studies of recent U.S. Oversight involving agricultural biotechnology

We chose three case studies representing the first gene-edited food products cleared for the U.S. market: the first plant-based product designed for improved oil (high-oleic acid soybean); the first animal-based gene-edited food product (heat-tolerant cattle); and the first whole-food vegetable product designed for a less pungent taste (mustard greens). We first collected information about the products from the peer-reviewed literature and other sources, and then analyzed the regulatory process and documents regarding their regulatory clearance. Finally, we looked at the regulatory processes and assessments in light of the parameters stakeholders and consumers identify as important (Table 1; Figure 2). These examples are provided in the subsequent sections to give an indication of the emerging risk and regulatory review processes for gene edited agrifood products in the U.S. to help identify the strengths and shortcomings of oversight and suggest improvements for the future.

### 3.1 Soybeans with altered oil composition

This case study was chosen because it is the first gene-edited crop available in the market. In 2015 a gene-edited soybean line with increased levels of oleic acid and decreased levels of linoleic acid was cleared by the USDA through its “Am I Regulated” process (USDA, 2015a; b), which was in place from 2010 to 2020 prior to the SECURE rule being implemented (USDA, 2020a). Potential benefits of increasing the levels of oleic acid in soybean include benefits to food manufacturers, as higher oleic soybean oil provides higher heat stability and may extend product shelf lives (Huth et al., 2015). Additional benefits includes serving as a potentially healthier replacement of saturated fats in foods to ones that may reduce risks of coronary heart disease (FDA, 2018). The company that produced this product, Calyxt, consulted with the FDA a few years later under the agency’s voluntary notification process for biotechnology-derived novel foods (FDA, 2019a; b, c). The product was generated using Agrobacterium-mediated transformation of TALEN site directed nucleases (gene-editing proteins that were precursors to CRISPR-Cas9) into the host soybean to make deletions in two FAD2 genes (USDA, 2015a; USDA, 2015b; FDA, 2019a; FDA, 2019b; FDA, 2019c). Then the transgenic sequences from Agrobacterium and the TALENs were backcrossed out to leave only the two deletions. As a result, USDA decided it did not have to go through its Plant Production Act regulations (prior to SECURE) (USDA, 2015a; b) as it did not contain DNA sequences from plant pests. Therefore, the plant did not have to undergo the plant pest risk assessment process or an environmental assessment under the National Environmental Protection Act. The decision document authored by USDA conveys the focus of the USDA determination as to whether the oil-altered gene-edited soybean is a regulated article (USDA, 2015b; Box 1). The focus of USDA’s determination is on the presence of plant pest sequences and that soybean plants are not considered plant pests. Weediness of soybeans was also considered, although it should be noted that weediness is not included as a primary risk endpoint in USDA’s regulations for genetically engineered plants (USDA, 2020a; Kuzma and Grieger, 2020).

Box 1 **Excerpt from determination that gene-edited soybeans are not regulated articles by USDA.**

“APHIS regulates the importation, interstate movement and environmental release (field testing) of certain genetically engineered (GE) organisms that are, or have the potential to be, plant pests. Regulations for GE organisms that have the potential to be plant pests, under the Plant Protection Act, are codified at 7 CFR part 340, “Introduction of Organisms and Products Altered or Produced Through Genetic Engineering Which Are Plant Pests or Which There Is Reason To Believe Are Plant Pests.” Under the provisions of these regulations, a GE organism is deemed a regulated article if it has been genetically engineered using a donor organism, recipient organism, or vector or vector agent that is listed in §340.2 and meets the definition of a plant pest, or that is an unclassified organism and/or an organism whose classification is unknown, or if the Administrator determines that the GE organism is a plant pest or has reason to believe is a plant pest. The TALEN and the other genetic sequences important to the development of the soybean were derived from plant pests….

According to your letter, the individual plant cells were genetically engineered to generate nucleotide deletions in two genes and thereby disrupt the function of specific proteins. However, all of the genetic material used to create the deletion was removed from the final soybean plant. Additionally, no genetic material was inserted into the final soybean plant genome. Based on the information cited in your letter, APHIS has determined this FAD2KO soybean variety was developed using [*removed due to Confidential Business Information*] and genetic material from plant pests. However, the final soybean plant does not contain any introduced genetic material and APHIS has no reason to believe that the plants of this soybean variety are plant pests. Therefore, consistent with previous responses to similar letters of inquiry, APHIS does not consider the FAD2KO soybean product as described in your 17 November 2014 letter to be regulated under 7 CFR part 340. Additionally, soybean is not listed as a Federal noxious weed under 7 CFR part 360, and APHIS has no reason to believe that the genetic engineering of your GE soybean would increase the weediness of soybean”.

Several parameters from Table 1 and Figure 2 are missing from this assessment including environmental impacts such as ecotoxicity, climate change impacts, resource use, and chemical use. Rather, USDA’s authority for GM plants is limited to plant pest risks under its GE plant regulations and the Plant Protection Act, and to a certain extent noxious weed risks under the PPA. This leaves several gaps for environmental toxicity and ecological sustainability that would only be considered under a broader Environmental Impact Statement under the National Environmental Policy Act (NEPA). EIS’s have been done for only a handful of decisions for GM plants in their 30 years history (see Kuzma, 2022) and NEPA analyses only take place when GM plants come under USDA’s plant pest risk authorities, which the gene edited soybean did not. It should be noted that the EPA has no authority for the gene-edited soybean as it did not introduce a “plant-incorporated protectant” or increase a pesticidal compound in the engineered plant (EPA, 2023). Some ecotoxicity parameters would have been considered under EPA’s FIFRA regulations for “plant-incorporated protectants” introduced or altered via genetic engineering (EPA, 2023).

As far as ELSI parameters and important parameters to consumers, transparency and stakeholder inclusion in the USDA decision making process was lacking. The Am I regulated? process under the former USDA plant pest regulations for GM crops involved letters published on the website and some of the information may be considered confidential business information (USDA, 2015a; b; Kuzma and Kokotovich, 2011; Kuzma, 2018; Kuzma, 2022). There was no publication in the Federal Register, no external advisory committee or external scientific input, and little risk or benefit information provided. Furthermore, the gene-edited soybean or oil derived from it would not need to be labeled under the National Bioengineered Food Disclosure Standards as there is no foreign DNA in the final product (Jaffe and Kuzma, 2021). This also means that consumers and other stakeholders will not be able to track where the product is being used in the marketplace and would remain unaware of it being gene-edited (Kuzma and Grieger, 2020).

As far as human health parameters are concerned for the gene-edited soybean, these would come under the FDA’s authorities under the FDCA. However, the FDA process is a voluntary consultation process which may decrease trust in consumers. Regardless, the company did take the step to consult with FDA and submitted information about the composition of the product in comparison to conventionally bred soybeans and oil derived from them for consideration by FDA for its suitability for food and feed (FDA, 2019a; b, c). These tests are generally designed to demonstrate nutritional “substantial equivalence” to the conventional counterpart. Endpoints in these documents that were considered include the fatty acid composition and its alteration; moisture, crude protein, crude fat, ash, and carbohydrates by calculation; fiber; amino acids, six fatty acids, three isoflavones (daidzein, genistein, and glycitein), four lecithins, and five anti-nutrients (lectin, phytic acid, raffinose, stachyose, and trypsin inhibitor) in whole seeds; and six fatty acids and lecithins. The FDA notes that “Calyxt states that the genetic modifications (inactivation of the FAD2-1A and FAD2-1B proteins, which are primarily expressed in developing seeds) do not meaningfully affect composition and nutrition of the meal derived from FAD2KO soybeans except for the intended changes in the levels of specific fatty acids” (FDA, 2019b). However, it is important to note that FDA relies on company data and does not make a determination of safety through this process, but states that it has “no further questions” (Box 2). These could reduce consumer trust in the oversight process. Although animal welfare, another important parameter to stakeholders and consumers (Section 2.1, 2.2), was not explicitly considered, impacts on animal health from consumption were according to the review of compositional data by FDA’s Center for Veterinary Medicine (CVM) (FDA, 2019b).

Box 2 **Excerpt from FDA’s consultation letters on gene-edited oil-altered soybean**

**“**Calyxt concludes:

• it has not introduced into food a new protein or other substance that would require premarket approval as a food additive
• food from FAD2KO soybean is comparable to and as safe as human food from other high oleic soybeans
• oil from FAD2KO soybean has a fatty acid profile consistent with criteria for “high oleic soybean oil"
• “high oleic soybean oil” is an appropriate common or usual name for oil from FAD2KO soybean

We evaluated data and information supporting these conclusions and considered whether FAD2KO soybean raises other regulatory issues involving human food under the Federal Food Drug and Cosmetic Act. We have no further questions at this time about the safety, nutrition, and regulatory compliance of food from F AD2KO soybean.”

The presence of nontarget edits was considered through Whole Genome Sequencing (WGS) and FDA states that the company found no evidence of new mutations in the seven genes with greatest similarity to the target sites. Although there is a low probability off target edits that would increase or decrease endogenous plant secondary compounds that may be allergenic or toxic to humans and animals, toxicity tests were not required. *A priori,* the product would not be expected to be any less safe for consumption than conventionally bred soybeans, however, unintended biochemical changes due to the change in the oil composition of the product or off-target edits outside of the seven genes with the greatest similarity could lead to a change in the toxicity or allergenicity profile of the product. There would be no way to determine the negligible health risk without whole food testing in animals or comprehensive metabolomic, proteomic, and gene expression testing (as suggested by the National Academies, see NASEM, 2016). The FDA review of gene-edited products and the company’s presentation of data are generally based on arguments about “substantial equivalence” yet based on macronutrients. In general, substantial equivalence is ill-defined and not a proxy for equivalent risk to conventional products (Millstone et al., 1999), as the limited nutritional and biochemical analyses done for FDA review may not account for unintended changes in the product (Cellini et al., 2004).

Another important set of parameters missing on the human health side are health benefits to consumers and impacts on food security and improved nutrition. The public has to rely on the company’s assessment that high-oleic acid soybean oil may be better for health than regular soybean oil from conventionally bred plants. FDA does not have a mandate to consider health benefits and claims from GM foods.

### 3.2 Heat tolerant cattle

The PRLR-SLICK cattle is the first gene edited animal to hit the market. In particular, CRISPR-based gene editing has been used in two founder beef calves to alter the prolactin receptor gene (PRLR gene) which shortens the prolactin receptor protein (PRLR protein) in cattle to obtain a short and slick haircoat (FDA, 2022). This intentional genomic alteration (IGA) is heritable and can therefore be passed to their offspring (FDA, 2022). However, the developed cattle are mosaic, therefore first-generation progeny may not all inherit the SLICK phenotype (FDA, 2022). The goal is to make beef cattle more tolerant to heat, similarly to several cattle breeds raised in the tropics which naturally developed this desired mutation as an adaptation response to the environment in which they have been bred (FDA, 2022). As reported in the FDA risk evaluation document, previous studies found that cattle with slick hair are more suitable for hot weather (FDA, 2022). In addition to improving heat tolerance, gene-edited slick hair cattle could also help expand cattle production to new areas as well as better adapting to increased temperatures related to climate change (Karavolias et al., 2021).

Although the slick mutation naturally occurs in some breeds of cattle, the use of gene editing makes the introduction of this mutation in other beef cattle breeds faster compared to traditional breeding, while also avoiding the loss of other desirable traits and potentially minimizing the introduction of undesirable traits (Parliamentary Office of Science and Technology, 2022).

In the U.S., the primary federal agency that regulates gene edited animals is FDA through the new animal drug provision of the Food, Drug and Cosmetic Act (FD&C Act). Section 201(g)(1)(C) of the FD&C Act contains the definition of a “drug”, which includes “articles (other than food) intended to affect the structure or any function of the body of man or other animals” (see 21 U.S.C. § 321(g)(1)(C)). Based on the definition of a “drug”, the genetic material inserted in the animals’ DNA that alters their structure or function falls under the drug definition of the FD&C Act (OSTP, 2017). According to the FD&C Act, any new animal drug needs prior approval from the FDA before being commercialized (OSTP, 2017). Genetically engineered animals with foreign genes, such as the AquaAdvantage Salmon, have been regulated under this act according to the 2009 FDA guidance #187 (revised 2015) to industry on the Regulation of Genetically Engineered Animals Containing Heritable Recombinant DNA Constructs (FDA, 2009; FDA, 2015). Under this guidance, a full Investigative New Animal Drug (INAD) or New Animal Drug (NAD) application was required (e.g., see Kuzma and Williams, 2023 for GE salmon; Kuzma, 2021b for GE mosquitos).

FDA put forth a new draft guidance in 2017 to include gene-edited animals under the FDCA, “GFI #187 Regulation of Intentionally Altered Genomic DNA in Animals” (FDA, 2017). Remarkably, in March 2022, the FDA used its enforcement discretion to review the PRLR-SLICK cattle under a less extensive approval process that did not require a full INAD or NAD, but produced a 8 page risk assessment summary authored by FDA. The agency made this first low-risk determination for enforcement discretion for a gene edited animal concluding in the risk assessment document that “there are no identifiable direct or indirect effects of the truncation of the *PRLR* gene or the IGA on the safety of food derived from the PRLR- SLICK cattle” (FDA, 2022, p 7). FDA also concluded that “the safety of food products made from PRLR- SLICK cattle is no different than the safety of food products made from commercial cattle that do not contain the IGA including those conventionally raised cattle with the naturally occurring slick phenotype” (FDA, 2022, p 7). As a result, the developers are not required to obtain FDA approval for a new animal drug prior to marketing the products derived from the gene edited cattle (Van Eenennaam and Mueller Maci, 2022). The FDA’s decision is limited only to those two founder cattle and their progeny (FDA, 2022).

It is important to note that this determination was made even though both the developer and FDA detected unintended, off-target mutations in the founder calves’ genomes (FDA, 2022). This is because the FDA determined that the types of unintended mutations and their positions would not change the protein expression compared to the non-edited cattle, although no data to demonstrate this was included in the risk assessment (FDA, 2022). Therefore, they were not considered as a risk for those that consume the products derived from these cattle (FDA, 2022).

As it relates to Table 1, the parameters considered for this product in the risk assessment include human health parameters such as the quality, nutrition, and safety of the SLICK cattle derived products. However, no data was shown in the risk assessment on the nutritional “substantial equivalence” of the beef from the cattle or toxicity or allergenicity in comparison to conventionally bred cattle, although conclusions of safety were made (FDA, 2022). FDA concluded that “conventionally raised cattle with the slick phenotype are routinely consumed as human food and therefore FDA does not expect a change in the compositional or nutritional content of the edible tissues derived from the PRLR-SLICK cattle because they are similar in genotype, phenotype, and health status of naturally occurring slick cattle. No hazards were identified that required further characterization” (FDA, 2022, p. 6-7).

In terms of food security and access, this product could be beneficial if beef production would be increased and more resilient from rises in global temperature which have already caused thousands of cattle deaths (Bushard, 2022). At the same time, an increased production and consumption of beef may potentially lead to a detrimental increase in environmental resources and land usage, especially if production is expanded to areas previously not suitable for cattle farming. This may also have adverse effects on climate change. Additionally, although there is unclear data on whether the SLICK cattle could lead to increased production and consumption, there is some data on adverse human health effects associated with high consumption of red and processed meat (World Health Organization, 2015). Data on these indirect implications for sustainability (such as land use, climate change, and agrochemical use in Table 1) were not explicitly included in the risk assessment, although a discussion of whether the cattle would escape and become feral was included in the risk assessment under “Environmental Risk” (FDA, 2022, p. 8). We recognize that these land use and consumption patterns may be hard to predict prior to market release of the cattle; however, they could be modeled under different scenarios upstream of market approval to inform post-market monitoring strategies for detecting these landscape changes and subsequent risk mitigation strategies (see Discussion).

Animal welfare and health are also other important parameters that need to be considered for gene editing in animals. Among the three calves with the IGA, one founder animal died unexpectedly due to a heart defect (attributed to bovine congestive heart failure; BCHF), although this was assumed not to be caused by the gene edits but a marker gene also present in the non-edited parents (FDA, 2022). Other aspects of the animals’ health were equivalent to non-gene edited comparator cattle (FDA, 2022). In fact, the welfare of cattle could increase because of this mutation, as those animals would tolerate higher temperatures better. At the same time, there is unclear data on the actual welfare of the SLICK cattle, meaning their emotional wellbeing and behavior in industrial living conditions is largely unknown. Although it is reported that the cattle’s nutrition, preventive health, and veterinary observation were representative of typical cattle production practices (FDA, 2022), the cattle subject to the evaluation were kept under rigorous physical containment and housing conditions and were not therefore observed in actual industrial farms conditions (FDA, 2022). This is a relevant knowledge gap because to assess whether the DNA changes affected animal welfare and health or to determine whether adjustments to the management, housing or nutrition are required, a wide set of measures as well as multiple indicators and a multi-disciplinary approach should be used (EFSA Panels on GMO and AHAW, 2012). For example, the European Food Safety Authority (EFSA) suggests a three-stage assessment of gene edited animals before commercialization. Stage A requires a laboratory-level monitoring of the intended effects of the edit and potential effects on the animals’ welfare through a set of health and welfare measurements, chosen between those established by the Welfare Quality^®^ project, that are tailored to assess the specific gene edit. Stage B requires an experimental farm assessment to assess the effects of the intended and/or any unintended effects of the gene edit on animals’ welfare in specified, licensed farms also called experimental farms. This stage would require a higher number of animals in order to observe the behavior of gene edited animals in relation to other animals. Finally, stage C requires a field trial in farms which practices are common across the European Union (EU).

Animal welfare and health are important parameters for stakeholders given that, and as highlighted by recent studies, consumers appear to be generally more supportive of gene editing applications in animals if those lead to increased animal welfare or health, while are generally less supportive of edits that focus on productivity traits (e.g., improved muscle tissue growth) (Yunes et al., 2021). However at the same time, gene editing may be viewed as a misguided substitute for conventional husbandry practices rather than meaningful welfare improvements. In fact, public opinion studies demonstrate that overall, there is less support for gene editing of animals compared to plant species, with ongoing discussions about the ethical and societal implication of gene editing in animals.

### 3.3 Less pungent mustard greens

This case study was chosen because it is the first whole vegetable product to be marketed for direct human consumption (i.e., without processing). Gene-edited mustard greens are expected to hit retailers and restaurants in late 2023 (Mullins, 2023). Researchers have gene-edited mustard greens (*Brassica juncea*) to remove the pungent and bitter flavors (Karlson et al., 2022; Grinstein, 2023). The potential benefits of developing gene-edited leafy greens include the ability for consumers to have access to nutritious leafy green products that taste better, which in turn, may increase consumption of healthy foods. Developers were able to do this by utilizing CRISPR to target and edit genes in order to reduce the production of oils made from glucosinolates that can cause a pungent taste when chewed or cut (Karlson et al., 2022). The genetic manipulation has significantly edited multiple genes across seven chromosomes of mustard greens, including the deletion of two whole genes, blocking the conversion of glucosinolates to these pungent oils.

In terms of regulatory oversight, the gene-edited mustard greens fall would conceivably fall under the jurisdiction of the USDA and the FDA according to the Coordinated Framework on Biotechnology Regulation (OSTP, 2017). However, in August 2020, USDA-APHIS determined that the gene-edited mustard greens do not fall under USDA’s regulations for genetically engineered crops as they do not contain plant pest DNA and thus do not pose a plant pest risk. This was determined as part of the *Am I Regulated?* process whereby the company sent a letter to USDA inquiring about the regulatory status of the gene-edited mustard greens, and USDA sent a response back as to whether the product would fall under its regulations under the Plant Protection Act (USDA, 2020c; USDA, 2020d; USDA, 2023). In the letter to USDA, the company noted that it “requests formal confirmation from USDA APHIS Biotechnology Regulatory Services (BRS) that *Brassica juncea* (L.) with improved flavor developed using gene-editing plant breeding tools is not a ‘regulated article’ subject to APHIS oversight under 7 C.F.R. part 340 because it will not contain any inserted genetic material from a plant pest” (USDA, 2020d). The company also described how no species of *Brassica* is listed as a Federal Noxious Weed and that the gene-edit would not be expected to make it into a weed. However, it should be noted that that certain *Brassica* species are considered weeds according to the USDA’s own weed risk assessments (e.g., USDA, 2021).

USDA cited the process of the modification and lack of plant pest DNA (and any foreign DNA left in the product) in their decision to exempt the gene-edited mustard from its regulations (USDA, 2020c). Although the USDA considered that the gene-edited mustard was not a plant pest and did not contain plant pest DNA, the assessment did not include investigations into other aspects of plant health such as the environmental consequences of removing genes involved in plant defense and the corresponding potential use of chemicals to control insects in the event of a pest outbreak. The gene-editing process changes glucosinolate metabolism in the plant and may deactivate the plant defense systems by blocking the metabolism of glucosinolate into insect-resistant components (Karlson et al., 2022). These metabolic changes could make the plants more vulnerable to insect pests under certain conditions, although no change was observed in the occurrence of insects in field trials of gene-edited mustard greens in a variety of locations and conditions (Karlson et al., 2022). In addition, environmental gene escape is a potential risk as gene-edited mustard greens may hybridize with other *B. juncea* or *Brassicas* (turnips) or may impact nearby related crops or weedy populations as well as surrounding ecosystems (e.g., such as non-target organisms). Information and data on the increased pest and weediness potential of the use of gene-edited mustard greens was not considered in the brief Am I Regulated letters. Also, toxicity to species in the environment from the biochemical changes in the gene edited mustard was not addressed in the brief *Am I Regulated* letter.

Shortly after USDA’s approval of the gene-edited mustard greens, the USDA’s Sustainable, Ecological, Consistent, Uniform, Responsible, Efficient (SECURE) rule came into effect at the end of 2020 (Hoffman, 2021). SECURE revised regulations for genetically engineered plants under USDA and the Plant Protection Act under 7 CFR part 340 (USDA, 2020a; Kuzma and Grieger, 2020; Hoffman, 2021). Under the current SECURE rule, the gene-edited mustard greens would also not likely be subject to regulation because the gene editing only deletes genes (USDA, 2020a).

The mustard greens also did not go through the formal, voluntary FDA consultation process^1^ and no Biotechnology Notification Files appear for the product on FDA’s website, although there are reports that the company consulted with FDA in a private meeting about the product (Mullins, 2023). This negated the investigation of any human health parameters in Table 1, including food safety and toxicity as it relates to the increase in glucosinolates. As it relates to the human health parameters in Table 1, the gene-edited mustard greens were developed to have a change in food quality that could also alter consumption patterns. It is anticipated that the less pungent mustard greens may promote the consumption of nutritious and healthy fresh produce, although no published data are available on this aspect. While pungency may currently prevent some consumers from eating mustard greens, the reduced pungency of their gene-edited counterparts may conceivably lead to unintended elevated exposures to glucosinolates when consumed in large amounts. This could become a health issue for vulnerable individuals who may be more impacted by such exposures.

The product would also not be subject to the National Bioengineered Disclosure Standards which mandate labeling as “bioengineered” or “derived from bioengineering” if a genetically engineered food product has foreign DNA in the final product (Federal Register, 2018; Jaffe and Kuzma, 2021). Given a lack of foreign DNA in the final food product from gene-edited mustard, it would not require labeling (Jaffe and Kuzma, 2021). In addition, much information in the company’s letter to USDA was deleted due to confidential business information (USDA, 2020d). Thus, parameters related to consumer transparency and stakeholder inclusion in Table 1 are lacking in the decision making process for this product. However, the developers of the mustard greens have conducted taste tests with consumers to better understand consumer preferences for the gene-edited greens and of gene-editing and CRISPR, and have pushed for transparency in the process of developing and applying this product by making it known publicly that its product is gene-edited. However, attention to many of the ELSI, health and environmental parameters is lacking in the mustard greens case with no FDA review, limited review by the USDA, and a lack of transparency to consumers more broadly.

## 4 Summary of the case studies

From the case studies above, we demonstrate that there are clear limitations for the federal agencies to consider many of the parameters that are important to consumers and diverse stakeholders and for assessing the sustainability of gene-edited agricultural products. For instance, in-depth environmental assessments were not required for either of the gene-edited plant crops (soybeans, mustard greens) as both were exempt from USDA’s plant-pest regulations for genetically engineered crops. Health assessments for the gene-edited soybean oil provided the most data on nutritional “substantial equivalence”, although toxicity studies were not conducted. Health assessments for the gene-edited mustard greens were not available and seem not to have been conducted under FDA’s voluntary consultation process. For the gene-edited animal product, the health assessment of the beef from gene-edited cattle was primarily qualitative, based on the assumption that the meat would be the same as meat from the non-edited cattle. Animal welfare for the gene-edited cattle was considered, although data was not presented in the assessment. Across all three case studies, broader parameters related to land, water and agrochemical use, and ecotoxicity were not evaluated for any of the products. Further, all agency approval processes were conducted without public or stakeholder input, and only between the product developer and federal agency. None of the products would require labeling under the National Bioengineered Food Disclosure Standards, and limited to no safety data is available to consumers. We also note that even when the assessment documents are available, they are difficult to find on federal agency websites. Overall, we argue that even if there are no obvious health or environmental safety concerns for these gene-edited products based on the available data and information, these aforementioned limitations will likely undermine consumer and public trust in gene editing and the arguments that these products will contribute to greater sustainability. We also note here that potential risks and limitations of these gene-edited agrifood products should be reviewed alongside their potential benefits. Holistic benefit assessments could be conducted in parallel to holistic risk assessments to create a comprehensive and balanced assessment of gene-edited agrifood products, taking into account health, environmental, animal health, and ethical and socio-economic factors. Multi-criteria Decision Analysis (MCDA) is one decision-support tool that may be particularly helpful to consider various benefits and risks of gene-edited agrifoods, and has been used in other food applications decisions when balancing benefits and risks (Ruzante et al., 2017).

## 5 Conclusions: Policy options

As demonstrated in the preceding sections, U.S. federal agencies that review gene-edited products are limited by their narrow regulatory authorities under current federal laws and the Coordinated Framework for Regulation of Biotechnology. For example, USDA is limited to “plant pest risks” and EPA is limited to “plant pesticide risks.” This creates gaps in what sustainability parameters can be assessed for novel agrifood technologies including products of gene-editing. In response, we propose several policy options for U.S. federal agencies to strengthen their oversight processes for agricultural biotechnology.

First, a broader assessment could be required through an Environmental Impact Statement under the National Environmental Policy Act. While no federal agency has exercised such an assessment for a gene-edited crop to date, a few have been done for genetically engineered crops with transgenes and therefore this may serve as a model to follow in future evaluations (see Kuzma, 2022 for details). In addition, federal agencies may have rather narrow regulatory scopes, although they can still require the minimum level of safety data for new gene-edited agrifood products, particularly those that are among the first to come to market. For instance, requiring at least nutritional “substantial equivalence” data or a minimal level of mammalian and non-target animal toxicity testing, and making such results available to consumers, would set the stage for greater consumer safety and trust. This policy recommendation would rely on a more open and comprehensive review process under existing regulatory processes rather than to require new institutions or legal foundations. The most rigorous and transparent process would also include open public advisory committees for decision making about certain gene-edited products and require Environmental Impact Statements under NEPA. At the same time, less rigorous improvements would include requiring more data and analysis for health and environmental safety under the current, closed regulatory processes (e.g., mandating the voluntary consultation process for FDA, assessing nutritional “substantial equivalence”, and requiring whole-food toxicity studies).

A second set of policy recommendations stemming from our analysis would require a novel institutional or cross-institutional framework. For example, federal agencies (or a trusted third party) could sponsor the development and ownership of a unified website (or database) for all gene-edited products on the market that are cleared for marketing by the federal agencies, which includes safety information, review documents, and potential market uses. This publicly-available website would also help improve transparency for diverse publics and other stakeholders in terms of better understanding which gene-edited agrifood products are currently on the market. The National Academies of Science, Engineering and Medicine in fact suggested a common portal of entry for biotech products to improve coordination of the federal agencies and avoid potential jurisdictional overlaps or gaps (2017). Further, Kuzma & Grieger (2020) suggested a repository like this for gene edited crops in order to improve public transparency and contribute to greater public choice and trust. In addition to a website or database, another option could be for a trusted third party research agency to do a more holistic sustainability assessment that would accompany each gene-edited product as it reaches the market place. Perhaps a research arm of the federal government or an independently funded think-tank could conduct such assessments and make them publicly available. The importance of this assessment is emphasized by the fact that several biotech developers argue that gene edited products will improve ecosystems, food security, and human health; and hence, it is important to back up these claims with a holistic assessment of the parameters in Table 1. A third party venue for these analyses could also improve public trust by showing that biotech developers’ claims are indeed legitimate. One such multi-stakeholder coalition to assess sustainability of gene edited cover crops has already been previously proposed and could serve as an example to move forward (Jordan et al., 2017). We do recognize, however, that upstream assessments for sustainability (e.g., landscape changes, consumption patterns) are likely to come with significant uncertainty and a lack of predictive power. In these cases, modeling can be used to consider impacts on sustainability under different use scenarios to inform decision making, post-market monitoring, and risk mitigation strategies, rather than as a regulatory checkpoint for initial market release. However, post-market surveillance mechanisms for biotech products in food and agriculture are currently limited under federal agency authorities (e.g., EPA for re-registration of plant-pesticides, FDA recall authorities for adulterated foods).

Finally, a third policy option that could be considered is developing a comprehensive, new biotechnology oversight law that requires the agencies to review each gene-edited product to some extent for a minimal set of health, environmental, and socio-economic variables. This is put forward given that there are several parameters included in Table 1 that are missing in assessments of gene-edited agrifood products including the investigated case studies in this work. For example, important environmental and human health parameters were missing from assessments in each of the case studies investigated, including mandatory food safety reviews (e.g., FDA’s process is voluntary, not performed for mustard greens case study) and environmental assessments (e.g., USDA’s authority is limited to “plant pest risk,” while ecosystem risks are outside the scope, including harm to nontarget organisms or indirect water or land use changes). Requirements for public transparency were lacking in all cases. Such a comprehensive oversight system with new legal authorities for genetically engineered products has in fact been previously considered (e.g., Kuzma, 2016; Kuzma, 2021a). Further, the National Academies of Science Engineering and Medicine also recently suggested a novel governance framework that will enable policymakers to better and anticipate and address the social, legal, ethical, and governance issues associated with emerging technologies as they arise (Mathews et al., 2022), although it is recognized that political will is needed for such approaches (Kuzma, 2023).

## Data Availability

The raw data supporting the conclusion of this article will be made available by the authors, without undue reservation.

## Footnotes

1 Biotechnology Notification File is available on FDA’s website, and in personal communication with the developer’s, it was confirmed that the product did not undergo that process. Instead, it was reported in personal communication that the company met with the FDA at some point prior to the press releases that the Conscious Greens would appear on the market. There is no evidence or content of that meeting available to the general public however.
